# Development of a Highly Biocompatible Antituberculosis Nanodelivery Formulation Based on Para-Aminosalicylic Acid—Zinc Layered Hydroxide Nanocomposites

**DOI:** 10.1155/2014/401460

**Published:** 2014-06-23

**Authors:** Bullo Saifullah, Palanisamy Arulselvan, Mohamed Ezzat El Zowalaty, Sharida Fakurazi, Thomas J. Webster, Benjamin Geilich, Mohd Zobir Hussein

**Affiliations:** ^1^Materials Synthesis and Characterization Laboratory, Institute of Advanced Technology(ITMA), Universiti Putra Malaysia, 43400 Serdang, Selangor, Malaysia; ^2^Laboratory of Vaccines and Immunotherapeutics, Institute of Bioscience, Universiti Putra Malaysia, 43400 Serdang, Selangor, Malaysia; ^3^Department of Environmental Health, Faculty of Public Health and Tropical Medicine, Jazan University, Jazan, Saudi Arabia; ^4^Department of Human Anatomy, Faculty of Medicine and Health Science, Universiti Putra Malaysia, 43400 Serdang, Selangor, Malaysia; ^5^Department of Chemical Engineering and Program in Bioengineering, Northeastern University, Boston, MA 02115, USA; ^6^Center of Excellence for Advanced Materials Research, King Abdulaziz University, Jeddah 21589, Saudi Arabia

## Abstract

Tuberculosis is a lethal epidemic, difficult to control disease, claiming thousands of lives every year. We have developed a nanodelivery formulation based on para-aminosalicylic acid (PAS) and zinc layered hydroxide using zinc nitrate salt as a precursor. The developed formulation has a fourfold higher efficacy of PAS against mycobacterium tuberculosis with a minimum inhibitory concentration (MIC) found to be at 1.40 *μ*g/mL compared to the free drug PAS with a MIC of 5.0 *μ*g/mL. The newly developed formulation was also found active against Gram-positive bacteria, Gram-negative bacteria, and *Candida albicans*. The formulation was also found to be biocompatible with human normal lung cells MRC-5 and mouse fibroblast cells-3T3. The *in vitro* release of PAS from the formulation was found to be sustained in a human body simulated phosphate buffer saline (PBS) solution at pH values of 7.4 and 4.8. Most importantly the nanocomposite prepared using zinc nitrate salt was advantageous in terms of yield and free from toxic zinc oxide contamination and had higher biocompatibility compared to one prepared using a zinc oxide precursor. In summary, these promising *in vitro* results are highly encouraging for the continued investigation of para-aminosalicylic acid and zinc layered hydroxide nanocomposites *in vivo* and eventual preclinical studies.

## 1. Introduction

Tuberculosis (TB) has remained lethal to humans for centuries and is of great public health concern. There were about 1.4 million human deaths from TB and about 8.7 million people infected in 2012 [1, 2]. TB is also the second greatest killer of humans in the world by a single infectious agent after HIV/AIDS [1]. The situation has become even more dire by the reemergence of multidrug resistant TB (MDR-TB) and in 2012, approximately 450,000 people developed MDR-TB and there was about 37% deaths of MDR-TB [1].

Chemotherapy of TB has been complicated by multidrug prescriptions, dosing frequency, longer treatment duration, and adverse side effects associated with anti-TB drugs [3, 4]. Since the drug development is lengthy, costly, and time consuming, it should not be surprising that no new anti-TB drug has reached the market in over 5 decades with the last anti-TB drug approved (rifampicin) in 1963 [3–5]. To cope with the TB epidemic, there is an urgency to develop new anti-TB formulations which can decrease dosing frequency, shorten treatment time (with little to no side effects), and maintain therapeutic concentrations in the human body for longer periods of time [3–6].

Improved drug delivery systems (DDS) are possibly the best solution for treating TB as they can improve drug bioavailability for longer time periods and release the drug in a sustained local manner to avoid toxicity [4, 7, 8]. DDS could protect the drug from physical, chemical, and enzymatic degradation inside the body and not let the drugs become exposed to the healthy tissues; therefore, they could decrease the side effects associated with the free drug [4, 9]. The DDS can target the diseased site and this can lead to better therapeutic results [8, 9].

Different drug delivery systems have been designed and developed for anti-TB drugs, namely, mesoporous silica nanoparticles, polymeric nanoparticles like poly-n-butyl cyanoacrylate, polyisobutylcyanoacrylate, poly(DL-lactide-co-glycolide) inhalable microparticles, large porous microspheres, and so forth [9–14]. But there are certain issues associated with each of these new material systems; some of them are not fully biocompatible, have poor serum solubility, and cause inflammation, cytokine release, cell damage, and so forth [4, 15]. In this manner, we propose a new formulation which should not possess such disadvantages. The layered double hydroxides (LDHs) are inorganic nanolayers with numerous nonbiological applications (such as serving as catalysts, flame retardants, and chiral separation materials) and have also been applied as a safe material for the removal of toxic waste from water [16–19].

Layered double hydroxides (LDHs) have emerged as excellent biocompatible nanocarriers for the sustained release and targeted transport of different pharmaceutical agents [8, 20–23]. LDHs have a structure similar to hydrotalcite with some of the divalent cations replaced with trivalent cations resulting in a positively charged material with brucite-like (magnesium layered hydroxides) sheets stacked over one another layer by layer [24–26]. The positive charge of the LDHs sheets is neutralized by counter anions [25, 27]. Zinc layered hydroxides (ZLH) also have similar characteristics although they do not possess any trivalent cations and anionic intercalation which may possibly be due to the hydrogen bonding between the anions and ZLH. ZLH have been widely applied for the delivery of different pharmaceutical drugs, namely, ellagic acid, hippuric acid, cetirizine, cinnamic acid,* m*-aminobenzoate, 2,4-dichlorophenoxy acetate, and so forth [20, 28–31]. Previously we have developed a sustained release formulation of anti-TB drugs based on para-aminosalicylic acid (PAS) with zinc layered hydroxides using zinc oxide as a starting material [7]. In this effort, we present the development of PAS-ZLH (termed nanocomposite A) using ZnNO_3_ salt as the starting material and compare the physicochemical as well as biological properties of such materials to the previously developed formulation using ZnO as the starting material (nanocomposite B) [7].

The nanocomposite-A is free from toxic zinc oxide contamination which is very difficult to avoid using a zinc oxide precursor. The nanocomposite-A (prepared using zinc nitrate salt) was found to be highly biocompatible compared to the nanocomposite-B (prepared using ZnO). In addition, the yield of the nanocomposite-A was also much higher compared to nanocomposite-B.

## 2. Materials and Methods

### 2.1. Materials

Para-aminosalicylic acid, 99% purity, and zinc nitrate hexahydrate were purchased form Sigma-Aldrich. Dimethyl sulfoxide (DMSO) was purchased from Ajax Fine Chem (Sydney, Australia). All of these chemicals were of analytical grade and were used without any further purification. Deionized water was used for all the experimental studies.

### 2.2. Preparation of Zinc Layer Hydroxides

Zinc nitrate hexahydrate was directly dissolved in 50 mL deionized water and the solution was stirred for 15 minutes under a nitrogen atmosphere. Then, the pH of the solution was increased by the slow addition of a 1 molar sodium hydroxyide solution with constant stirring under a nitrogen atmosphere.

### 2.3. Preparation PAS-ZLH (Termed Nanocomposite-A)

A 0.4 mol/L solution (50 mL) of PAS was prepared at a 1 : 1 ratio of DMSO and deionized water and was stirred for 15 minutes. Zinc nitrate hexahydrate, 1 gram dissolved in 50 mL of water, was stirred for another 5 minutes and after that the PAS solution was directly added to this solution. The sample was further stirred for 20 minutes. The pH of the final solution was raised to 7.9 by the dropwise addition of a 1 molar sodium hydroxide solution and then the solution was further stirred for 1 hour. The whole experiment was conducted under continuous nitrogen flow. Finally, the sample was subjected to an oil bath agitation at 70°C for 18 hours. The sample was centrifuged, washed three times, dried at 70°C, and ground to fine powder for further characterization as described below.

### 2.4. Material Characterization

X-ray diffraction (XRD) analysis was carried out on a Shimadzu (Kyoto, Japan) XRD-6000 Diffractometer. XRD patterns were recorded in the range of 2 *θ* = 2–60°, at the Cu*K* *α* radiation at 30 kV and 30 mA. Fourier-transform infrared (FTIR) spectra of samples were recorded in the range of 4000–499 cm^−1^ by the direct sample method with a PerkinElmer (Waltham, MA, USA) 100 series spectrophotometer. For the elemental analyses of carbon, hydrogen, nitrogen, and sulfur (CHNS), a LECO (St Joseph, MI, USA) CHNS-932 instrument was utilized. For the thermogravimetric and differential thermogravimetric analyses, a Mettler-Toledo (Greifensee, Switzerland) instrument was used. The sample surface morphology was captured with a JEOL (Tokyo, Japan) JSM-6400 scanning electron microscope (SEM). For optical properties and controlled-release studies, a Shimadzu 1650 series (Japan) UV-Vis spectrophotometer was utilized. The percentage of the PAS loading was determined using a Sykam HPLC system with a Sykam S3250 UV/Vis detector, an auto injector Sykam 5300, and Sykam quaternary pump system 5300 made in Germany, with a column Zorbax Rx-Sil 4.6 × 150 mm, with 5 *μ*m particle sizes (Agilent). For the quantification of the metallic element zinc, an inductively plasma (ICP) optical emission spectrometer (an Optima 2000 DV, Perkin Elmer) was used. Particle size of the nanocomposite was determined with a dynamic light scattering (DLS) technique by using a Zeta sizer nanoseries—NANO-S Malvern instrument.

### 2.5. Controlled-Release Study

The release behavior of PAS from nanocomposite-A was studied in a human body simulated phosphate buffer solution (0.1 mol/L) at a pH of 7.4 and a pH of 4.8. about 0.3 mg of the nanocomposite was placed in a 3 mL quartz cell and then was placed in a UV/Visible spectrophotometer. The lambda max of PAS (268 nm) was selected for the UV/Vis spectroscopic studies [2].

### 2.6. Bacteria Studies

#### 2.6.1. Antimicrobial Sensitivity Test

The drug susceptibility test (DST) of nanocomposite-A was determined using a nonradiometric fluorescence-based method of MGIT 960 against* Mycobacterium tuberculosis *(ATCC 25618). The mycobacteria growth indicator tube (MGIT) with a BACTEC MGIT 960 growth supplement for drug susceptibility testing (DST) was used in the MGIT 960 instrument (Becton Dickinson Diagnostic Systems, Sparks, MD, USA) as described previously [32, 33]. The standard protocol for DST in MGIT 960 was strictly followed as recommended for primary drugs. Culture suspensions for inoculation were well dispersed with no large clumps to avoid false-resistant results.

After thorough mixing and homogenization of the culture suspensions, the tubes were allowed to rest for at least 15 min, and the supernatant was used to inoculate the drug-containing media and the control according to the manufacturer's instructions for DST of first-line drugs. All inoculated drug-containing MGIT 960 tubes were placed in the DST set carrier and entered into the MGIT 960 instrument as “unknown drugs” using the DST entry feature. For the DST set containing “unknown drugs,” the instrument flagged the DST set “complete” when the growth control reached a growth unit (GU) value of 400. At that point, the GU values of drug-containing tubes were retrieved from the instrument by printing out a DST set report, and results were interpreted manually. If the GU of the drug-containing tube was more than 100 when the GU of the growth control was 400, the results were defined as resistant. If the GU values of the drug-containing tubes were equal to or less than 100, the results were considered susceptible. Experiments were repeated with various concentrations of PAS nanocomposite suspensions until the respective MIC values were determined.

(*1) Nonmycobacterium Antimicrobial Susceptibility Testing.* The PAS-ZLH (nanocomposite-A) was tested for its antimicrobial activity against different microorganisms including* Staphylococcus aureus *(ATCC 43300)*, Pseudomonas aeruginosa *(ATCC 27853)*, Escherichia coli *(ATCC 25922), and* Candida albicans *(ATCC 20408) and were purchased from the American Type Culture Collection [ATCC], Manassas, VA, USA using the standard plate colony counting method. Percentage inhibition of the nanocomposite was plotted as previously described [34].

### 2.7. Cell Studies

#### 2.7.1. Cell Culture

Human normal lung MRC-5 and mouse fibroblast 3T3 cells were bought from the American Type Culture Collection (ATCC; Manassas, VA, USA), and the cells were cultured in Dulbecco's modified Eagle's medium (DMEM) and RPMI 1640 media containing 10% fetal bovine serum (add the manufacturer information). Growth media contained 100 units/mL of penicillin and 50 *μ*g/mL of streptomycin. Fibroblasts were maintained at 37°C in a humidified atmosphere in the presence of 5% CO_2_.

#### 2.7.2. Assessment of Cytotoxicity by the MTT Assay

Healthy cells were seeded onto 96-well culture plates at 1 × 10^4^ cells per well and were allowed to adhere overnight at 37°C. Then, the cells were incubated with the above medium (100 *μ*L) containing dispersed LDH nanocomposites in various concentration ranges from 0.781 *μ*g/mL to 50 *μ*g/mL. The control cells were not exposed to the nanocomposites. At specific time points of 24, 48, and 72 hours of incubation, the growth medium was removed from the 96-well plates and incubated with 100 *μ*L of the MTT (3-(4,5-dimethylthiazol-2-yl)-2,5-diphenyltetrazolium bromide) reagent in DMEM for another 3-4 hours at 37°C. The number of viable cells was analyzed by the uptake of MTT and read at 570 nm by an enzyme-linked immunosorbent assay plate reader. Cell viability results were presented as the mean ± standard deviation.

### 2.8. Statistical Analysis

Unpaired* t*-tests were used to compare the MICs of PAS and nanocomposite-A against* Mycobacterium tuberculosis*. Statistical analysis was used to compare the percentage inhibition of PAS and nanocomposite-A against different microorganisms using two way-ANOVA tests. The Prism V6.01 statistical software (GraphPad, San Diego, CA, USA) was used for data management and statistical analysis. ANOVA followed by student* t*-tests were used to determine the differences between the means of cell viability (%). All data are shown as the mean ± standard deviation unless indicated differently.

## 3. Results and Discussion

### 3.1. X-Ray Diffraction (XRD) Analysis

**Figure 1:**
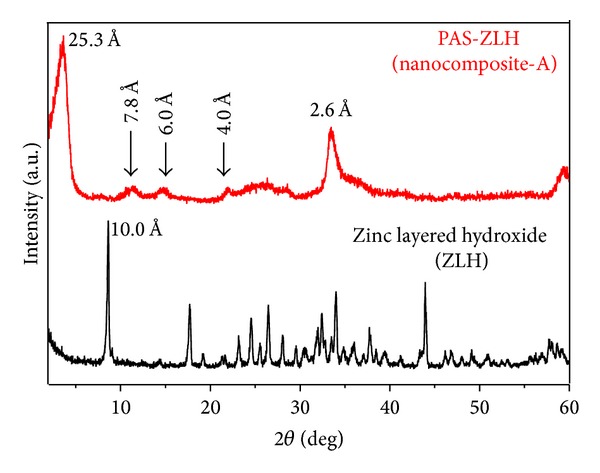
X-ray diffraction pattern of zinc layered hydroxide (ZLH) and PAS-ZLH (nanocomposite-A).

Figure 1 shows XRD patterns of the zinc layered hydroxide (ZLH) and PAS-ZLH (nanocomposite-A) materials. In the XRD pattern of the ZLH, there are many small peaks with a first major peak with high intensity at about 2*θ* = 9.0° with basal spacing of about* d* = 10 Å (Figure 1), corresponding to nitrate counter anions as due to the reflection of the 200 planes of the monoclinic structure and is also consistent with a previous report [35]. In the XRD pattern of PAS-ZLH (nanocomposite-A), the increase in basal spacing from 10.0 Å to 26.0 Å is strong evidence for the successful intercalation of PAS into the interlayer galleries of ZLH (Figure 1).

In addition to the first reflection (with a* d*-spacing of 26 Å), PAS-ZLH also showed four more reflections with* d*-spacing values 7.8 Å, 6.0 Å, and 4.0 Å which indicates the high crystallinity of the nanocomposite. There is a small hub from 2 *θ* = 22° to 28° which can be ascribed to the adsorption of PAS on the ZLH surface, as the area coinciding with XRD peaks of free PAS has prominent peaks in that region, as reported previously [7]. The nanocomposite-B prepared using zinc oxide (ZnO) as a starting material showed five characteristic peaks of ZnO, due to the 100, 002, 101, 102, and 110 planes between 2 *θ* of 30–60, which indicates the presence of unreacted ZnO [7]. However, the current formulation prepared using ZnNO_3_ as the starting material did not contain any ZnO. In addition, the yield of the nanocomposite-A was much higher compared to the one using ZnO as the precursor (nanocomposite-B).

### 3.2. Spatial Orientation of PAS in the Interlayer of ZLH for Nanocomposite A

**Figure 2:**
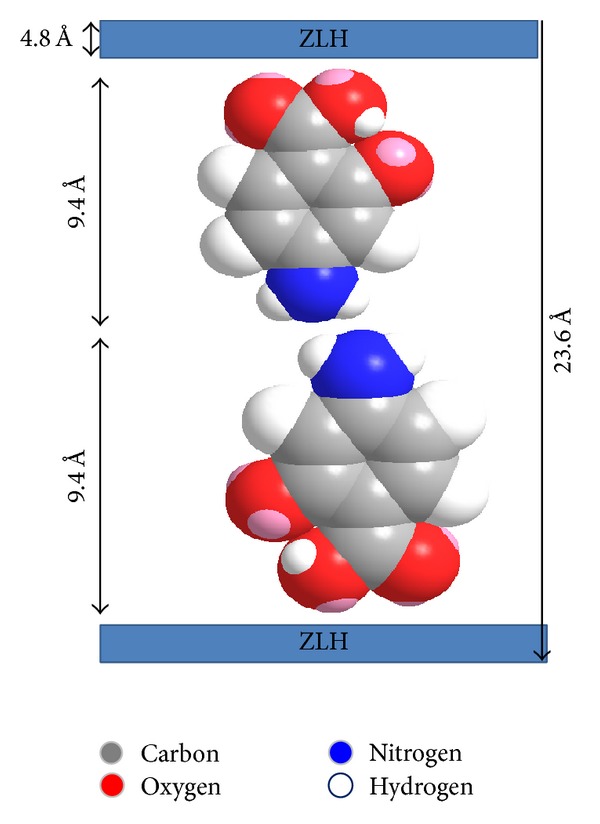
Spatial orientation of PAS in interlayers of ZLH (nanocomposite-A).

Based on the 3-D molecular size of PAS, the* x*,* y,* and* z* axes of PAS have been reported to be 9.4 Å, 7.1 Å, and 2.9 Å, respectively [7], and the thickness of the ZLH layer has been reported to be 4.8 Å [35]. The average basal spacing for the nanocomposite-A was found to be 23.6 Å; by subtracting the layer thickness ZLH (4.8 Å), a value of 18.80 Å was obtained. This value of 18.8 Å strongly suggests that PAS has been oriented in a bimolecular vertical form (*x*-axis) as shown in Figure 2. The PAS-ZLH prepared using ZnO has been reported to have a bimolecular horizontal (*y*-axis) orientation of PAS into the interlayers of ZLH along with water molecules [7].

### 3.3. Infrared Spectroscopy

**Figure 3:**
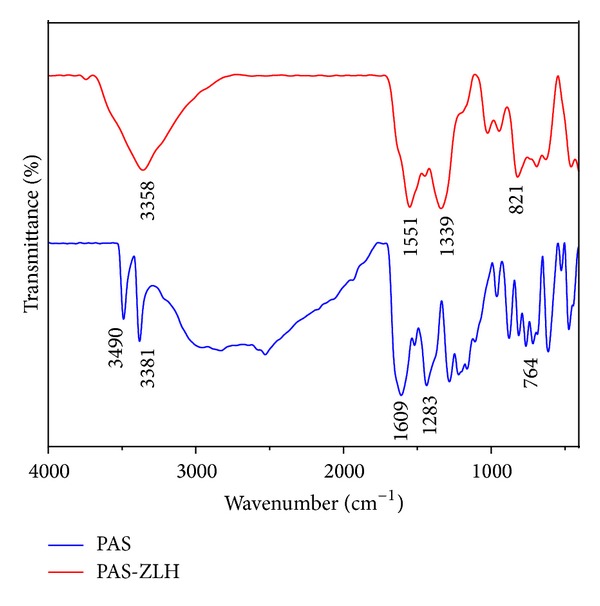
FTIR spectrum of PAS-ZLH (nanocomposite-A).

Fourier transform infrared (FTIR) spectroscopy gives information about the presence, absence, and shifting of bands of functional groups and can be very useful in supporting other analytical results. FTIR spectrum of pure PAS showed the characteristic bands for its functional groups at specified positions as carbonyl at 1609 cm^−1^ and symmetric and asymmetric N–H bands at 3381 and 3490 cm^−1^, respectively; others are given in Table 1 [7]. In the spectrum of PAS-ZLH (Figure 3), N–H symmetric and asymmetric bands have been overlapped by the O–H band of the interlayer ZLH. In addition, the C=O band of PAS also disappeared instead of the two new bands which appeared at 1551 and 1339 cm^−1^ due to the symmetric and asymmetric bands of carboxylate (COO–), respectively. Most of the other FTIR bands of PAS are present in the nanocomposite-A with slight shifts in wavenumbers and are given in Table 1. The presence of characteristic bands of pure PAS and ZLH in the nanocomposite-A further confirms the XRD result of successful PAS intercalation into ZLH. The FTIR spectrum of the PAS-ZLH nanocomposite-A is similar to the one prepared using the ZnO nanocomposite-B [7].

### 3.4. HPLC Analysis

HPLC analysis of the active drug PAS in the nanocomposites was carried out by the methods reported by Hong et al. (2011) and Vasbinder et al. (2004) [36, 37]. In brief, the mobile phase of methanol as solvent A and solvent B phosphate buffer is composed of a 17.5 mM equal amount of monobasic and dibasic potassium phosphate of pH 3.5. The isocratic mobile phase of solvent A and solvent B at a ratio of 60 : 40 was used with a flow rate of 1 mL/minute. The wavelength of 233 nm was selected for detection using the UV/Vis detector. A calibration curve was obtained by running standards at different concentrations in parts per million (ppm) of PAS (i.e., 0.0 ppm, 20 ppm, 40 ppm, 60 ppm, and 80 ppm) with a good *R* ^2^ value of 0.98. Approximately, 10 mg of the nanocomposite was dissolved in 50 mL (5 mL of 1 molar HCl and the remaining volume was composed of the mobile phase) and the standard PAS solutions were also prepared in the same way. The retention time of PAS was found to be 1.8 minutes. The percent loading of PAS in the nanocomposite-A was found to be 22.24%. The loading in the nanocomposite-A (22.42%) was higher as compared to the nanocomposite-B (14.60%) reported previously [7].

### 3.5. Elemental Analysis

The presence of carbon and nitrogen was determined by CHNS analysis and the metal element zinc was determined by an inductively coupled plasma spectrometer. Elemental analysis of PAS and PAS-ZLH (nanocomposite-B) was taken from our previous report [7]. The presence of organic elements in the samples like carbon, nitrogen (N), and zinc further supported the XRD results for the successful intercalation of PAS into ZLH. The percentage of the each element is given in Table 2.

### 3.6. Thermogravimetric Analysis

**Figure 4:**
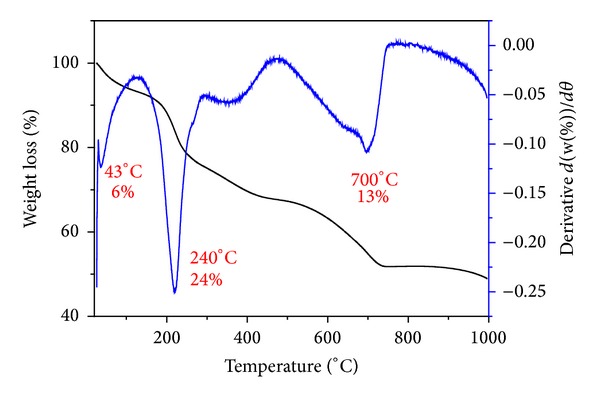
Thermogravimetric analysis-differential thermogravimetric thermograms of PAS-ZLH (nanocomposite-A).

The free drug, PAS, was thermally decomposed at 221°C with a percent mass loss of 67.7%, as reported previously by our group [7]. In the nanocomposite-B, PAS was stabilized and thermal decomposition shifted to 248°C [7].  Figure 4 shows the TGA analysis of the nanocomposite-A prepared by using ZnNO_**3**_ instead of ZnO as the starting material. There are three main mass loss events that took place for nanocomposite-A. The first event of mass loss of about 6% at 43°C can be attributed to the physically adsorbed water. The second event is the weight loss of 24% at about 240°C, which is due to the thermal decomposition of PAS. PAS has been thermally stabilized from 221°C to about 240°C; the increase in stability can be attributed to the electrostatic interaction between PAS and ZLH. The 3rd event occurred at about 700°C with 13% mass loss, and this can be attributed to the dehydroxylation of ZLH interlayers [20, 29]. The TGA analysis results of nanocomposite-A are almost comparable to the nanocomposite-B reported previously with only slight shifts in temperature and percentage mass loss [7].

### 3.7. Surface Morphology

**Figure 5:**
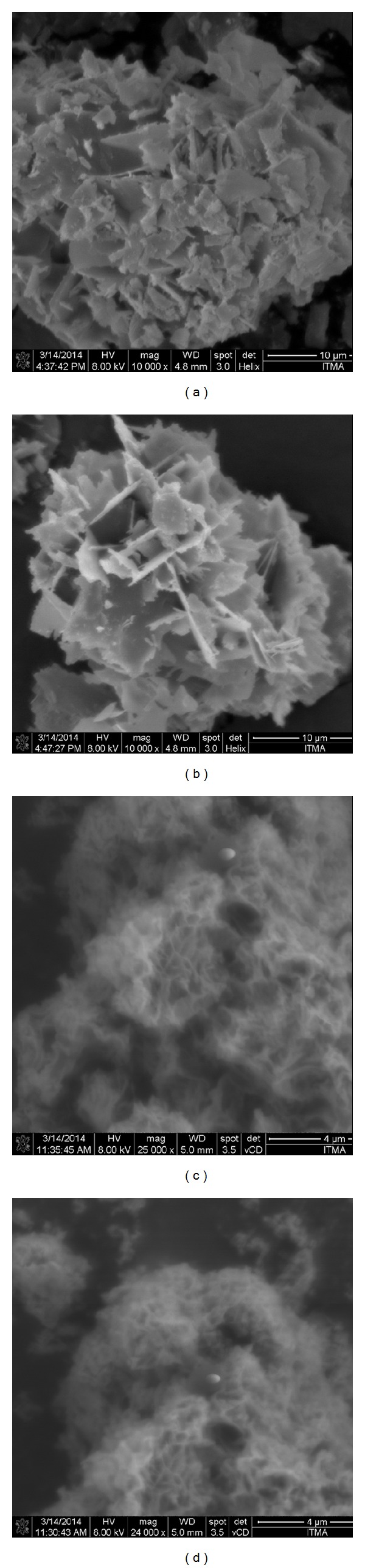
FESEM micrographs of ZLH ((a) and (b)) and PAS-ZLH (nanocomposite-A) ((c) and (d)).

Figures 5(a) and 5(b) show the surface morphology of the carrier zinc layered hydroxide (ZLH) with zinc nitrate intercalated. The morphology of ZLH is a 2-dimensional layer type as shown in Figures 5(a) and 5(b). The morphology of the nanocomposite PAS-ZLH is of honey comb type and the sample looks more compact (Figures 5(c) and 5(d)) unlike ZLH alone. The general ZLH have been reported to have plate-like, compact nonuniform agglomerate and rod-like agglomerate like morphology [7, 20, 28]. But for the ZLH nanocomposite this honeycomb morphology is rare; however, it has been reported previously for LDHs nanocomposites by Chen et al. (2012) [38].

### 3.8. *In Vitro* Release

**Figure 6:**
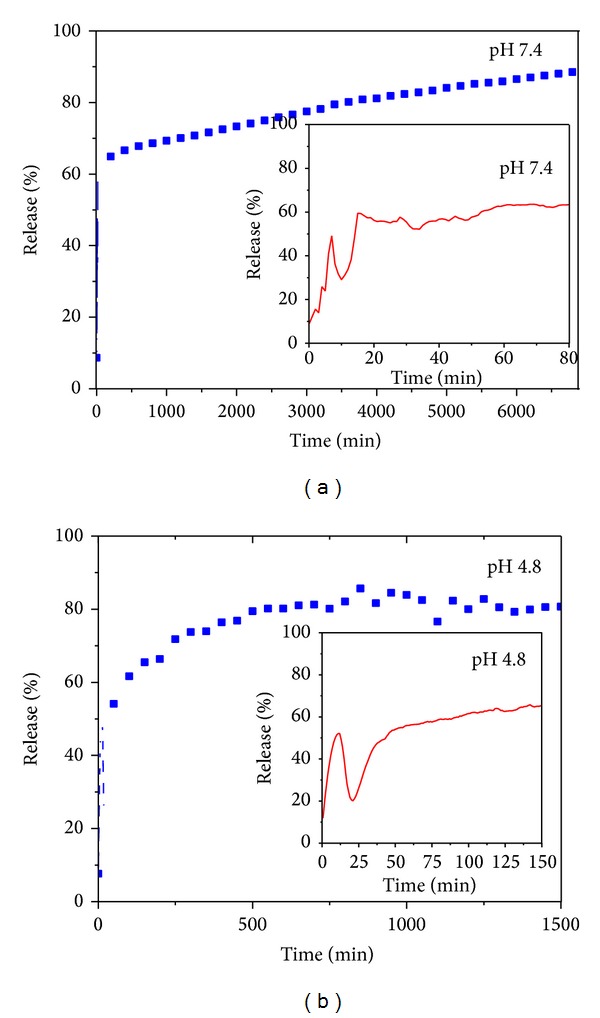
(a)* In vitro* release of PAS from PAS-ZLH (nanocomposite-A) in human body simulated phosphate buffer solutions of pH 7.4. (b)* In vitro* release of PAS from PAS-ZLH (nanocomposite A) in human body simulated phosphate buffer solutions of pH 4.8.

Figures 6(a) and 6(b) show the* in vitro* release of PAS from nanocomposite-A in human body simulated phosphate buffer solution (PBS) at pH values of 7.4 and 4.8, respectively. For the initial first 30 minutes, there is faster release of PAS followed by a much more sustained release up to 7000 minutes in PBS at pH 7.4 as shown in Figure 6(a), with an overall release of 94%. Similar trends in release were observed in PBS at pH 4.8, with an overall release of 99%. However, the overall release time was much shorter at pH 4.8 (i.e., 1500 minutes) as compared to PBS at pH 7.4 (i.e., 7000 minutes). The small inset on Figures 6(a) and 6(b) shows the initial release of PAS from the nanocomposite-A. The faster release at pH 4.8 can be ascribed to the different release mechanisms. At lower pH values, LDH released the drug by two phenomena, namely, (a) ion exchange and (b) weathering/degradation of the LDH inorganic layers by protonation [8, 39]. At the higher pH 7.4, the drug was released by ion exchange mechanisms only and not by weathering [8, 39]. These release trends of nanocomposite-A are similar to the nanocomposite-B as reported previously by us [7].

### 3.9. Release Kinetics of PAS from Nanocomposite-A

Three different kinetic models, namely, pseudo-first order, pseudo-second order, and parabolic diffusion kinetic equations, were applied to analyze the release kinetics of PAS. The model equations applied are described in standard form as below.

The pseudo-first order kinetic equation in the linear form can be described as

(1) $$\begin{array}{c} \ln q_{e}-q_{t}=\ln q_{e}-K_{1}t, \end{array}$$

where *q*_*e*_ is the amount released in equilibrium and *q*_*t*_ is amount released at time (*t*). *K*_1_ is the constant whose value can be determined from the slope by plotting ln⁡(*q*_*e*_ − *q*_*t*_) against *t* [40, 41].

The pseudo-second order equation is given below in its linear form [26, 42]:

(2) $$\begin{array}{c} \frac{t}{q_{t}}=\frac{1}{K_{2}q^{2}}+\frac{t}{q_{e}}. \end{array}$$

The standard parabolic equation can be described as follows:

(3) $$\begin{array}{c} \left(\frac{1-\left(M/M_{o}\right)}{t}\right)=k_{t}^{-0.5}+b, \end{array}$$

where *M*_*o*_ and *M*_*t*_ represent the amount of drug that remained in ZLH at release time 0 and at time *t*, respectively, and *b* is a constant [26, 43].

**Figure 7:**
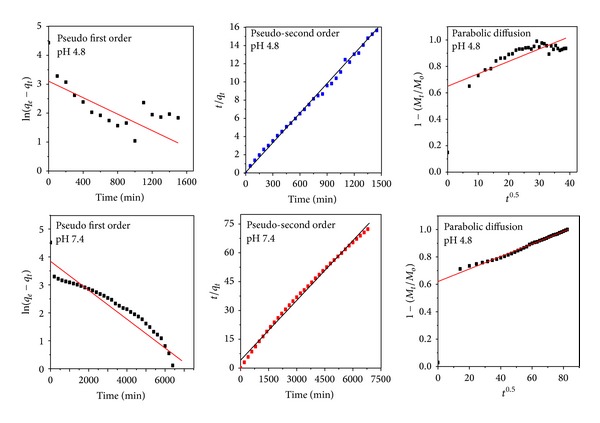
Kinetic fitting data for PAS* in vitro* release from PAS-ZLH (nanocomposite A) into PBS solutions at pH 7.4 and 4.8 by applying the pseudo-first and pseudo-second-order kinetics and parabolic diffusion model.

By applying the above three kinetic equations, we found that the release kinetics of PAS followed the pseudo-second order as the value of the correlation coefficient *R* ^2^ was greater than that for the other two equations both at pH 4.8 and pH 7.4, as given in Table 3. Table 3 also contains the rate constant value for the pseudo second-order model PAS release kinetics at pH 4.8 and 7.4.  Figure 7 represents fitting plots for the release kinetics of PAS released from nanocomposite-A at pH 4.8 and 7.4 obtained by applying all these three different kinetic models. We can clearly observe a straight line that is only obtained in the pseudo-second order model, which further suggests that the release kinetics follow the pseudo-second order route.

The pseudo-second-order release kinetics is actually a third order reaction which means the PAS release depends on the three things. Here, it can possibly be the concentration of the nanocomposite, the concentration anions present in PBS (i.e., HPO_4_^−2^), and the PBS medium environment.

### 3.10. Particle Size Analysis

**Figure 8:**
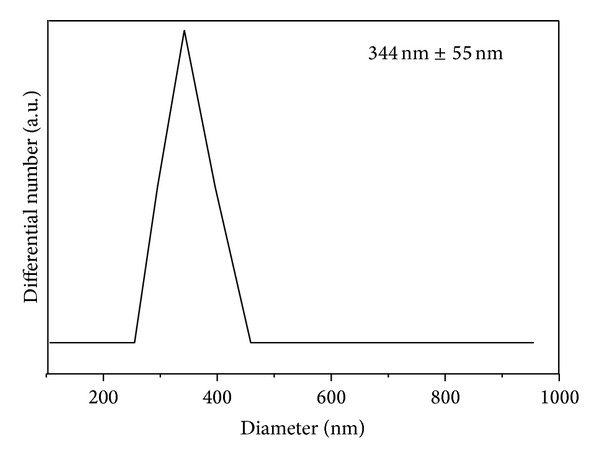
Hydrodynamic size of the PAS-ZLH nanocomposite-A.

The average hydrodynamic size of the PAS-ZLH (nanocomposite A) was determined by using dynamic light scattering (DLS) by using a zeta sizer. The sample was dispersed in deionized water and sonicated for 15 minutes. In order to get more accurate results, samples were measured three times. The average diameter of the nanocomposite-A was found to be 344 nm** ± **55 nm as the DLS graph shows in Figure 8.

### 3.11. Antimycobacterial and Antimicrobial Assays

**Figure 9:**
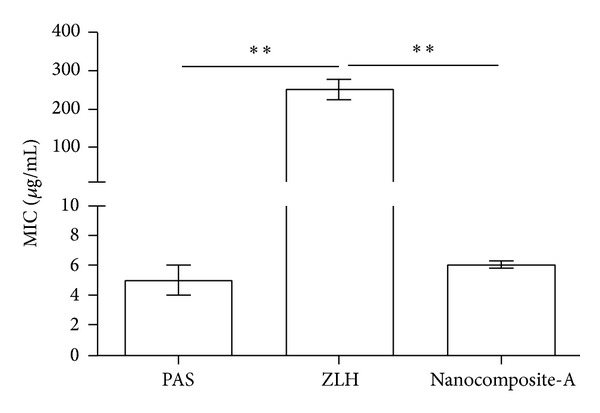
Minimum inhibitory concentrations (*μ*g/mL) (MICs) of PAS-ZLH (Nanocomposite A) as compared to PAS against* Mycobacterium tuberculosis* determined by the mycobacteria growth indicator tube (MGIT) with BACTEC MGIT 960 growth supplement for drug susceptibility testing (DST) and measured by the MGIT 960 instrument (Becton Dickinson Diagnostic Systems, Sparks, MD, USA).

**Figure 10:**
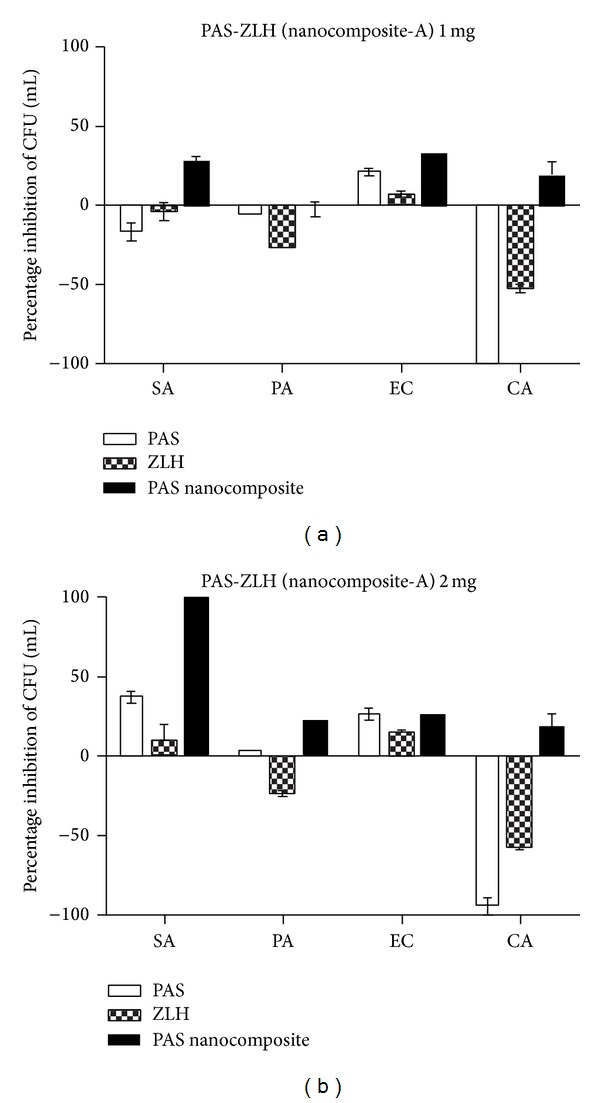
Effect of PAS-ZLH (nanocomposite-A) on the inhibition of microbial growth using the plate colony counting method at two concentrations ((a): 1 mg) and ((b): 2 mg). CFU: colony-forming units; SA:* Staphylococcus aureus*; PA:* Pseudomonas aeruginosa;* EC:* E. coli*; CA:* Candida albicans*.

The MIC of the PAS-ZLH (nanocomposite A) against* Mycobacterium tuberculosis* was found to be 6.1 *μ*g/mL as compared to that of the free drug (PAS) which was 5.0 *μ*g/mL as shown in Figure 9. The effective MIC concentration of the drug PAS in nanocomposite-A based on loading 22.42% is 1.40 *μ*g/mL which is about 4 times less than free drug PAS with an MIC of 5.0 *μ*g/mL. Based on this effective MIC concentration we can see the efficacy of PAS is fourfold higher when given in nanocomposite form compared to that given as free PAS. This improved efficacy of PAS in PAS-ZLH (nanocomposite A) can be attributed the nanoscale size and sustained release of PAS for longer periods time. The results of the antimicrobial testing found that the nanocomposite-A showed antibacterial activity against Gram-positive and Gram-negative bacteria and* Candida* as shown in Figures 10(a) and 10(b) from the percentage inhibition of each compound against different organisms. It was found that the nanocomposite was more active against Gram-positive (*Staphylococcus aureus)* and Gram-negative (*E. coli*) bacteria than* Pseudomonas aeruginosa *and* Candida albicans.*

### 3.12. Cytotoxic Study

#### 3.12.1. Cytotoxic Study of the Nanocomposite-A against Mouse Fibroblast Cells 3T3

**Figure 11:**
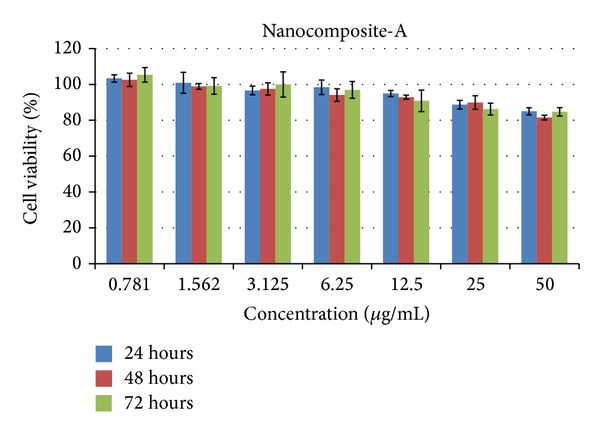
PAS-ZLH (nanocomposite-A) against mouse fibroblast cells 3T3.

Cytocompatibility of the nanocomposites was determined using a colorimetric method by the MTT assay.  Figure 11 shows the MTT assay results obtained using various concentrations (0.78 *μ*g/mL to 50 *μ*g/mL of the nanocomposite-A) against mouse fibroblast 3T3 cells for different period of times, that is, 24, 48, and 72 hours. The percent cell viability of nanocomposite-A was found to be very high, about 85% even at the highest concentration 50 *μ*g/mL for the longest duration of 72 hours. In comparison to nanocomposite-A, nanocomposite-B was reported to be biocompatible until 25 *μ*g/mL was added only for 24 hours and for 48 and 72 hours, 25 *μ*g/mL was biocompatible with about 80% cell viability [7]. However, at higher concentrations of 50 *μ*g/mL, nanocomposite-B was found to be cytotoxic as reported earlier [7]. Thus, we conclude that nanocomposite-A has higher biocompatibility compared to nanocomposite-B, even at higher concentrations for 72 hours using mouse fibroblast cells.

#### 3.12.2. Cytotoxic Study of Nanocomposite-A and Carrier Zinc Layered Hydroxide (ZLH) against Human Normal Lung Cells MRC-5

**Figure 12:**
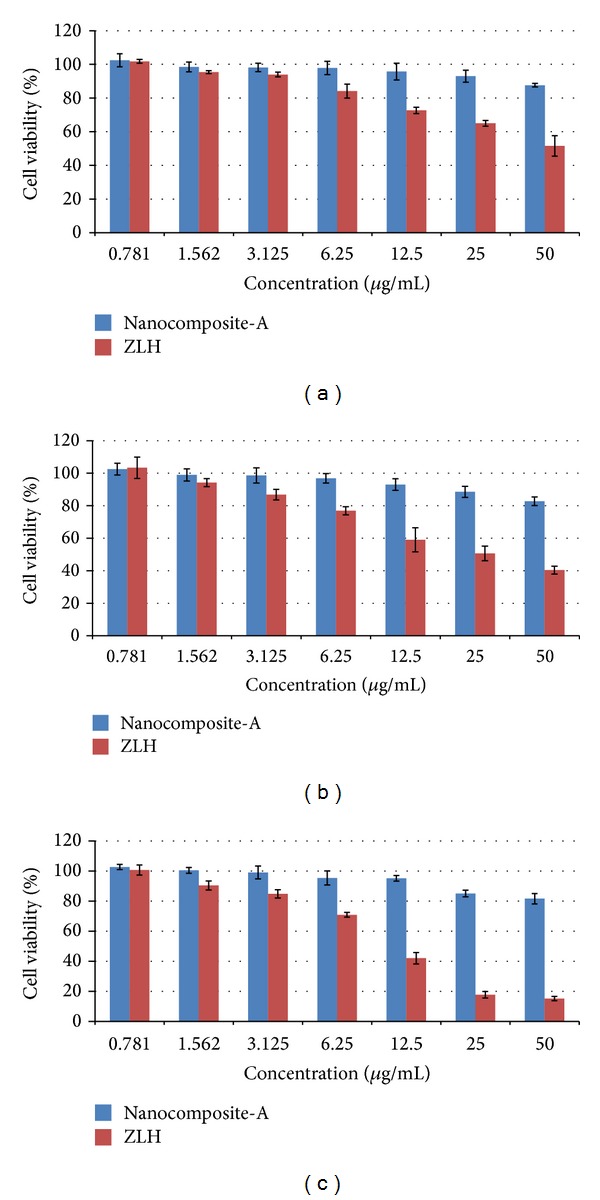
(a) 24-hour lung cells MRC-5 PAS-ZLH (nanocomposite A) and the carrier ZLH. (b) 48-hour lung cells MRC-5 PAS-ZLH (nanocomposite-A) and the carrier ZLH. (c) 72-hour lung cells MRC-5 PAS-ZLH (nanocomposite-A) and the carrier ZLH.

The most common form of TB is called pulmonary TB, where the bacteria reside in the lungs [44]. It is highly advisable for any new anti-TB formulation that its biocompatibility should be assayed against human lung cells. We conducted a cytotoxic study of the developed nanocomposite-A and the carrier ZLH against human normal lung cells, MRC-5. In order to check the biocompatibility, the MTT assay protocol was followed and different concentrations of the nanocomposites-A and ZLH ranging from 0.78 *μ*g/mL to 50 *μ*g/mL were treated against human normal lung cells MRC-5 for 24, 48, and 72 hours as shown in Figures 12(a)–12(c). The carrier, ZLH, itself was found to be toxic which may be due the presence of nitrate anions which are present as counter anions. However, nanocomposite-A was found to be highly biocompatible with cell viability at about 80% at the highest concentration of 50 *μ*g/mL for the longest treatment duration of 72 hours.

## 4. Conclusions

The present study describes the development of an antituberculosis nanodelivery formulation based on para-aminosalicylic acid with zinc layered hydroxides using ZnNO_**3**_ salt as a precursor.

The PAS* in vitro* efficacy was found to be fourfold better when used in the developed formulation compared to the free drug PAS. The formulation was also found active against Gram-positive bacteria, Gram-negative bacteria, and* Candida*. The PAS-ZLH showed very good biocompatibility with human normal lung cells and MRC-5 as well as with mouse fibroblast 3T3 cells. Furthermore, the* in vitro* release study of PAS from the interlayer galleries ZLH was found to be sustained in human body simulated phosphate buffer saline solutions of pH 7.4 and 4.8. In comparison to the previous PAS-ZLH, prepared using ZnO as the starting material, the current formulation gave better yield, loading, and biocompatibility properties. In summary, these promising* in vitro* results are highly encouraging for the continued investigation of para-aminosalicylic acid and zinc layered hydroxide nanocomposites* in vivo* and eventual preclinical studies.

## Figures and Tables

**Table 1:** FTIR functional group absorption bands of free PAS and its PAS-ZLH (nanocomposite-A).

Assignments	Free PAS	PAS-ZLH
Vas(N–H)	3490	Overlapped by O–H stretching 3358
Vs(N–H)	3381	
V(O–H) in the inter ZnLH, H_2_O	—	
V(C=O) in COOH	1609, 764	—
VAS(COO–)	—	1551
Vs(COO–)	—	1339
Stretching (C–H)	813, 717	821

Unit for given numbers is wave number (cm^−1^).

**Table 2:** Elemental analysis of organic and inorganic elements (nanocomposite-A).

Sample	C (% w/w)	N (% w/w)	C/N	Zn (% w/w)	% Loading of PAS by HPLC
PAS (pure drug)	59.00^a^	10.00^a^	6.02^a^	—	—
PAS-ZLH Nanocomposite-A	16.00^a^	2.30^a^	8.20^a^	44.62^b^	22.24^c^
PAS-ZLH Nanocomposite-B	10.54^a^	1.54^a^	6.8^a^	52.7^b^	14.60^c^

^a^ Determined by CHNS analysis; ^b^Determined by ICP analysis; ^c^Determined by HPLC analysis.

**Table 3:** Rate constant (*k*) and correlation coefficient *R* ^2^ determined from the release kinetics of PAS from PAS-ZLH (nanocomposite-A) into PBS solutions of pH 7.4 and pH 4.8.

Samples	pH	Release/%	*R* ^2^			Pseudo-second order
	—	—	Pseudo-first order	Pseudo-second order	Parabolic diffusion model	Rate constant *K*_2_ (mg/min)
Nanocomposite-A						
PAS-ZLLH	4.8	99	0.56	0.99	0.66	2.85 × 10^−4^
PAS-ZLLH	7.4	94	0.81	0.99	0.95	2.70 × 10^−5^

Of PAS are similar to the nanocomposite-B as reported previously by us [7].
